# “Without social there is no health”: Social work perspectives in multidisciplinary healthcare

**DOI:** 10.3389/fsoc.2022.1017077

**Published:** 2022-11-16

**Authors:** Roberta T. Di Rosa

**Affiliations:** Dipartimento Culture e Società, Università degli Studi di Palermo, Palermo, Italy

**Keywords:** social work, hospitalization, reintegration processes, community work, interprofessional recognition

## Abstract

The pandemic has not just affected the health sphere: strong social effects of the emergency have added to the health risk, stressing on social relations and the deterioration of people's living conditions, and making those who are already fragile more fragile. Notwithstanding, during the emergency following the COVID-19 pandemic the attention was focused, indeed understandably, on the health aspects, widening the already existing misalignment between the health interventions and the social ones. Emergency oriented efforts and resources more toward a clinical care approach (cure) than toward support for the social and the inclusion aspects (care). Reflecting on the specific area of health care that interacts with social care (and vice versa), shows how the medicalization in managing the emergency have undermined or, at least, weakened the global approach to the person and to vulnerability profiles that should inspire the socio-healthcare integration. The aim of this review is describing the relationship between the health and social systems and the effects of the COVID-19 pandemic on it: a review of studies on the role played by social work in the health sector before and during COVID-19 pandemic emergency shows how much potential there is still to be developed for social work in the health sector that acts together with the personal health services; a care that looks at the person within his or her relationships, community resources and environmental aspects requires an investment toward integration between hospital care, social services and local communities.

## Introduction

In a pandemic situation, the urgency of medical care is compounded by the need for social care, i.e., specific attention to personal, family, and neighborhood relations—despite social distancing—, careful and in-depth monitoring of the population's needs, facilitation of access to home care and specialist services, and a thorough social epidemiology study that integrates with the health one. Emergency affects an entire community, as well as the many personal and family life stories that result in individual emergencies, and require an immediate, or at least timely, social work intervention. The pandemic has not just affected the health sphere: as it is enshrined in the very definition given by the WHO, an all-encompassing conception of health is also extended to the social dimension. The risk of neglecting the social aspects should not be underestimated: suffice it to think of the needs related to support in daily life and the problems arising from social isolation and loneliness. Every hospitalized patient, in addition to being ill, suddenly found himself alone, without being able to count on family members who were strictly banned from the wards or simply from the same room. And into that void the social workers intervened, stemming the loneliness of patients, and helping their families (CNOAS, 2020). Moreover, social workers act as a link between health services, local authority services and the private social sector. They take care of people's connections, and (they) can be a bridge between the inside and the outside of the Intensive care, knowing how to work in difficult conditions, involving all spheres of a person's life (Allegri and Di Rosa, 2020).

The aim of this review is describing the relationship between the health and social systems, through a literature search on the current state of the relationship between social and health care, and to say that the pandemic has forced the hand with respect to the need for integration that until recently was little considered, making the urgency of its implementation evident today. “Hospitals are ground zero for pandemic” (Muskat et al., 2022, p. 129) and an important setting for social work practice, where during pandemic, social workers have been called upon to response to change and challenges in health care, to respond to increases in patient medical complexity.

Furthermore, it is proposed to interweave looked at integration prospects from the perspective of a community-based approach. The continuity of care requires an intervention that goes beyond the focus on the individual and his or her close circle of life. Various studies show that the more effective and sustainable pathways are over time insofar as social intervention on specific cases is combined with a broad-based commitment to the community (Cook, 1994). Wishing to explore the implications of professional methods connected with the commitment to “continuity of care,” it appears necessary to frame the appropriate type of intervention within the framework of actions defined in the literature as a “community development”. Studies by Reynolds (1982), Spergel (1987), Christenson and Robinson (1989), Twelvetrees (1991), Biggs (1999), and Driu (2014) can be recalled in this direction. Community development is a concept that currently has wide appeal in public health policy, as central element of population-based health promotion strategies that purport to involve community groups in determining the form and purpose of resources for advancing the community's health (Petersen, 1994). As Reynolds (1982) suggest, the survival of the community depends on its ability to meet the social development and social welfare needs of its residents. That's the reason because social work in community development acts as “a deliberate intervention into the social network or structure of relations among people and organizations in a local area or interest community to facilitate social problem solving and improve patterns of service delivery and sociopolitical functioning” (Spergel, 1987, p. 300).

## Studies and research about social work in COVID time: An overview

The COVID-19 pandemic impacted the knowledge production. Researchers and scholars wanted to make their contribution through scientific action, and from the very beginning of the pandemic they set in motion pathways to analyze the response to the emergency, seeking both to understand what the most relevant critical issues were for the present (Banks et al., 2020), and what could then be the significant elements to be recorded for future professional developments (Ben-Ezra and Hamama-Raz, 2020; Sanfelici et al., 2020; Ravalier et al., 2021; Fronek and Rotabi-Casares, 2022).

The contemporary academic debate is concentrated in observing and analyzing on pandemic experience to integrate social work knowledge at many levels. A high attention has been paid to innovative practices of support people, helping them deal with failing health systems and aftermath of widespread disease on families and communities (López Peláez et al., 2020; Mishna et al., 2021). Also, the discussion focuses on the need to work to change systems at the macrolevel and to value social work as an essential contributor to health emergency plans (Jen et al., 2021).

Focusing on the complex and difficult work conditions social workers faced during pandemic, an interesting line of research was that which revisited existing studies on social work in disasters and emergencies, reworking them by considering the specific (and absolutely new) case of a global pandemic (Harms et al., 2020; Biddle et al., 2021; Borenstein et al., 2021). The extraordinariness of the situation reflected on which strategies and methodologies were most effective; the results of more than one research converged on highlighting the importance of modifying social work job demands in disaster work, by learning from case examples of earlier disasters to prepare guidelines/recommendations for such events. A positive work environment with the re-assurance of personal safety during the COVID-19 pandemic were the main factors that were the key to encourage medical staff to continue working during the epidemic (Campisi et al., 2022).

Different directions of innovation were identified: among them, one considers the internal need of organizations and services, relating to the dynamics between professionals. Levin-Dagan and Strenfeld-Hever (2020) explored the experiences and strategies of hospital social work in Israel in responding that accompanied distanced social support for patients and families, taking in count the increase in mental health and grief support, as well as mediating heightened feelings of loss of control were adapted through virtual modalities. Muskat et al. (2022) explored the role changes, the availability of resources needed for the new job duties, emergent training needs with a survey conducted in December 2020 in Ontario. They developed interesting recommendations for pandemic social work practices in hospitals through the reorganization of hospital social service. In particular, they underline the centrality that emerges with respect to the need on the one hand for recognition for social service as “essential hospital staff” on par with other hospital professions, and on the other hand for collaboration between hospital administrators and social workers in building “clear protocols to ensure seamless transitions during future pandemic” (Muskat et al., 2022, p. 136) that take into account both social and medical assessments toward a shared readiness and flexibility.

This includes also adapting practice through innovative solutions (e.g., available technology), to determine which cases can be supported remotely and which require in-person visits, and how to ensure social worker workforce safety from contracting the virus (Cadell et al., 2022). The intersection between the social and health spheres, in the COVID-19 pandemic era, was represented using digital technologies as a privileged means to build and maintain a relationship among professionals, patients and families, despite distance, and/or isolation. In the post-hospitalization phase, psycho-social counseling “at a distance” (Sanfelici, 2020) was fundamental to guarantee guidance and information on the help available, psycho-social support and listening to the experiences of uncertainty, anguish and sometimes pain and suffering linked to the impact of the illness, interviews to support the elaboration of mourning, to people in isolation and quarantine, and to families called upon to reorganize care management after hospitalization.

Another strand of innovation is that of investment in the dynamics between services and the community in the direction of ensure meaningful collaboration and co-production with people with lived experience in health and social care (Biddle et al., 2021). While, the health system perspective is mainly focused on understanding COVID-19 pandemic physiological aspects, the different ways in which patient have re-integrated society and the support they have received from medical professionals after intensive care. Borenstein et al. (2021) stressed how the chosen critical and participatory approach helped them to create nuanced knowledge on the complexities related to the experiences of formal kinship care—something required and longed for by people who use services and practitioners alike.

Other authors, focusing the role of social work in health care at the intersection of these two directions of innovation—call for a spreading awareness of the relevance of the link between social and health care intervention (Fronek and Rotabi-Casares, 2022). With respect to the recognition issue, it had been highlighted yet before the pandemic (Wong, 2018) that gaining a place at the hospitals does not mean that gaining professional recognition. Moreover, literature shows that interdisciplinary approach has many barriers to overcome (Hua, 2004). Some notable ones include turf protection, different values and perceptions regarding problems and needs of patients, self-promotion, prestige, and status discrepancies that prevents open communications, skills and knowledge areas, and differences in problem solving processes (Cowles, 2003). To win recognition from medical, nursing, and allied health disciplines for contribution of social workers on the bedsides of patients requires a pathway that starts with formal and legislative recognition but needs to turn into cultural change and an appreciation of the relevance of the role and profession and its complementarity to health care pathways (Sen et al., 2020). Dealing with the changes taking place in pandemic time in the paths of recognition and enhancement of social service in health care, permits to observe that—even if recognition of government toward the importance of healthcare social work is often more formal than substantive—pandemic called social work upon to be the bridge between the inside and the outside of health care settings.

In this direction, then, it is appropriate for social workers to operate by activating and supporting community members to identify and take collective awareness, empowering, and resourcing the community members to create stronger and more connected communities (Twelvetrees, 1991). The social worker, in order to act as a liaison agent between the hospital and the territory must invest his or her cognitive effort and professional action both internally and externally, to ensure the activation of resources for each individual patient in connection with the “uniqueness of the community” (Driu, 2014) of reference. The concrete benefits of community development (…) came through local people changing attitudes, mobilizing existing skills, improving networks, thinking differently about problems, and using community assets in a new way (Tan, 2009).

## Italian debates and experiences

With respect to the social work response in Italy, there has been no shortage of studies that have stressed, since the first lockdown period in 2020, the impact of emergency in social work practices and had have given attention to the operational processes at work, analyzing their characteristics and developments throughout the pandemic period (Allegri and Di Rosa, 2020; Binkin et al., 2020; Dellavalle and Cellini, 2020; Sanfelici et al., 2020; Terraneo, 2020; Cabiati, 2021; Fargion et al., 2020). In the Italian debate, much emphasis has been given precisely to the need for a paradigm shift in the management of public health emergencies: to combat the epidemic effectively, a community-based approach appears as the model that can best address the social and health needs related to the pandemic, while being aware of the difficulties of this profound transformation of the health system (Dente, 2020). The reference to a community-based approach finds its meaning in the reaffirmation of social capital as a community resource, in the paths of rediscovering “solidarity.” A solidarity-based response is the only one that can quickly address the effects of inequality amplified by the pandemic: “Building socially and individually acceptable systems of solidarity will be the great challenge of the coming times. (…) Solidarity, then, is the only ‘social' response, easily achievable for all” (Lo Verde, 2022, p. 83).

Italian authors (Giarelli and Vicarelli, 2020; Terraneo, 2020; Pirrone, 2022) have been immediately reading the pandemic experience in the broader context of the already existing vulnerability of healthcare system (Gori, 2017; Smorto, 2020). Italy's response has been characterized by some rapid measures to tackle the health crisis, but few plans in the mitigation stage and a lack of community involvement. The impact on society, in terms of pre- and post-hospitalization social vulnerability, has made the absence of a national policy capable of building an adequate system of long-term care, the shortcomings of home care, the imbalance on monetary transfers, the scarce support and valorization of caregivers, the marginal role attributed to social services, often understood as mere providers of benefits. The model centered on hospital care did not prove to be the most suitable to manage the effects of a pandemic, if it was not adequately supported by territorial health services and prevention activities on the ground (Nacoti et al., 2020).

Although in Italy the role of the social worker as a connector of the clinical and care pathways should be better enhanced—particularly for the continuity of care between the hospital and the territory—, the pandemic stimulated the official recognition of the centrality of sociomedical intervention. The community dimension of Social Work in Health System was stated in 2010 by the Italian Ministry of Health, where it was stressed the objective of integration between hospitals and territories, to be achieved through specific actions as networking all the resources in the territory, ensuring integrated and synergistic interventions, acting as a promoter of strategies for rationalization and integration between the health and social system. It enables the implementation of an intervention model based on a multidimensional and integrated concept of health, thanks to the professional specificity inherent in the training of the Social Worker and the profession's own ability to connect all areas of welfare (Italian Ministry of Health, 2010).

The public health measures issued in Italy during 2020 and 2021 focused on the more specifically health-related aspect, even if a willingness emerged to manage the emergency with specific attention to the social dimensions connected to the health ones, thus broadening the concept of emergency and intervention from the clinical to the social sphere. The measures enacted took this potential into account and introduced innovations in terms of professional recognition and inclusion in care settings, therefore constituting the start of a significant reform of services integration, e.g., with the institution of special units of “continuity of care” (named USCA) for the purposes of the multidimensional assessment of patients' needs and of the integration with the territorial social and socio-health services, that were brought into the system of integrated social and health services.

Some specific elements should be emphasized with respect to the Italian reality with a view to strengthening the role of social service within hospital care pathways. Social listening and helping relationship complement the action of doctors in filtering access to services, especially those with limited availability, and explaining why this is necessary, as well as directing people to other social, health and welfare services; as well as explaining and interpreting government policies, so that these are accessible to all segments of the population. Pandemics do not know about class differences, but they have an impact, accentuating even more inequalities and social injustices (Mazzola, 2022). The professional tasks assigned to social workers are, firstly, the assessment of emerging needs, the information on rights and orientation to services, and the listening and psycho-social support. Not less important, they work for guarantee to patients support and guide of their natural support networks, activating social capital (Putnam, 2000) both in sense of “bonding” and “bridging.” In the first one, organizing and coordinating the timely response to basic needs (distribution of food, drugs, finding safe housing, restoring safety conditions for health) and to relational needs, through the activation of formal network services (services for the elderly, for minors, for addictions, for victims of violence, for mental health) and informal network services (activation and networking of voluntary organizations that intervene to satisfy basic needs (home delivery). In the second one, building bridges between patients and their communities, through an involvement of the community in the analysis of emerging needs (with particular attention to less visible or less empowered groups), in the identification of resources to respond to these needs, in awareness-raising and information projects about health emergencies. In the case of the pandemic emergency, the activation of community resilience becomes an essential condition for the resilience of individuals, for the recovery and reintegration of post-COVID patients into a community that must rethink itself and the rules of coexistence and solidarity (Christenson and Robinson, 1989).

The presence of social workers can also make it possible to promote public health by mobilizing communities with respect to prevention, with a community education action, offering help to people in identifying how to keep themselves safe and how to practically live out the social distancing indications, even if it has been observed that the applicability of the community approach may be limited to countries where public health and curative services are integrated (Binkin et al., 2020). In addition to participating in efforts to strengthen health and social services as essential protection against the virus, social care also requires special attention to be paid to existing social services and a re-organization of work to enable services to remain open and proactive in supporting vulnerable communities and populations.

These lines of social action are intertwined with other essential functions: data collection on emerging needs with particular reference to groups or to the most vulnerable communities, constant updating of the map of the network of formal and informal social and health services present in the community of reference useful for dealing with the emergency; advocacy actions to give voice to the rights of the most vulnerable and most difficult to access people; experimentation with innovative solutions (use of digital technologies) to respond in a timely manner to emerging needs, ensuring an exchange through the national network.

Finally, the COVID-19 pandemic has confirmed how social and health integration is essential for the definition of integrated pathways for taking charge of citizens on the health and social fronts, even in emergency situations such as the one that occurred in the current context or on other occasions (post-earthquake or other similar events) and how the role of the social worker is strategic in guaranteeing an important function of connection, integration, and support. The action of the social worker proves to be indispensable, complementary and strategic to the transition from a condition of “extraordinary” to one of “everyday;” acting as a “ferryman” of the patient toward the achievement of a new equilibrium, aimed at favoring and facilitating the course of therapeutic and care continuity also at the Health and Social Services of the Territory after the exit or the overall needs with integrated projects shared, as was recently recognized in Ministerial Decree No. 77/2022 on models and standards for the development of territorial care in the national health service.

## Perspectives

This contribution stresses the importance of a cultural shift in health care system toward a stronger professional integration with social work, particularly with a community social development approach to build concrete actions of collaborative (Craig et al., 2020) and integrated practices in care settings (Sanfelici, 2020). As emerged clearly, the support of professional social workers can improve the capacity of the health system to respond adequately in terms of long-term community wellbeing, integrating health services with a broader range of information, prevention, and support activities for the population (Maglajlic and Ioakimidis, 2022). Social workers are the link between health services, local authority services and the private social sector. They take care of people's connections, as bridges between the inside and the outside the health system; never before integration between social work and health care have been as fundamental as they are now, in making care projects that cover all spheres of a person's life. The intervention on individuals cannot disregard an overall intervention on the social fabric to which the individual belongs, if one wants to work toward the restoration of lifestyles, the satisfaction of needs and the activation of resources and potential—necessary to enable people to regain confidence in social life and to be able to cope with difficulties, contributing to the redefinition of everyday life. As stated in the main global policy frameworks to guide disaster management (Pyles, 2007; Dominelli, 2015), the effort to achieve integration through project sharing in multi-professional teams and the implementation of effective interventions may overcome sectorial fragmentation and promote an ecological vision of the person and the living environment.

In conclusion, the outcome of the review presented is offered for the future consideration of scholars and professionals, with the aim of highlighting how the role of the social worker as a connector of the clinical and care pathways implemented in response to the COVID-19 pandemic emergency needs to be better defined and enhanced, for the continuity of care between hospital and territory. It is more necessary than ever to devise, together with hospital and territorial social and health professionals, new integrated procedures for the continuity of care pathways of protected discharge in the post-acute phase. As well, the review shows that there is potential to invest in, in the direction of strengthen cooperation between health and social services, paying a special attention to a re-organization of procedures in both fields, so that they serve, in a coherent and coordinated way, as open and proactive resources in supporting vulnerable communities and populations.

## Author contributions

The author confirms being the sole contributor of this work and has approved it for publication.

## Conflict of interest

The author declares that the research was conducted in the absence of any commercial or financial relationships that could be construed as a potential conflict of interest.

## Publisher's note

All claims expressed in this article are solely those of the authors and do not necessarily represent those of their affiliated organizations, or those of the publisher, the editors and the reviewers. Any product that may be evaluated in this article, or claim that may be made by its manufacturer, is not guaranteed or endorsed by the publisher.
